# Impact of Remedial Exercise Training on Work-Related Musculoskeletal Morbidity Among Handloom Weavers in Kanchipuram District: A Quasi-experimental Study

**DOI:** 10.7759/cureus.67217

**Published:** 2024-08-19

**Authors:** Stalin R, Denny Mathew John, Selvaraj I

**Affiliations:** 1 Community Medicine, Saveetha Medical College and Hospital, Saveetha Institute of Medical and Technical Sciences, Saveetha University, Chennai, IND

**Keywords:** work-related musculoskeletal morbidity, handloom weavers, remedial exercise training, occupational health hazard, quasi-experimental study

## Abstract

Introduction: The handloom weaving industry is integral to developing countries, especially in South Asia, where traditional techniques are still widely practiced. In India, the handloom sector is a significant part of the informal economy, employing millions and preserving rich cultural heritage. Despite its economic and cultural importance, the sector faces severe challenges, including poor working conditions that lead to a high prevalence of work-related musculoskeletal disorders (WMSDs). This study focuses on assessing the prevalence of musculoskeletal morbidity and the effectiveness of physiotherapy interventions in reducing WMSDs among handloom weavers in Kanchipuram, Tamil Nadu.

Methods: This quasi-experimental study utilized a pre- and post-test design conducted over 12 months. A total of 121 handloom weavers from four major cooperative societies in Kanchipuram were selected using multistage sampling. Inclusion criteria were adults over 18 years, full-time weavers with more than a year of experience, and those who consented to participate. The study involved initial data collection through interviews using a pre-tested semi-structured questionnaire and the Standardized Nordic Musculoskeletal Questionnaire to assess pain prevalence. The intervention phase included physiotherapy exercise training thrice a week for three months, followed by post-intervention data collection and analysis.

Results: Pre-intervention data indicated high prevalence rates of musculoskeletal pain, with 62% of participants reporting knee pain and 54.5% reporting ankle/foot pain over the past year. Post-intervention assessments showed significant reductions in pain across all body parts, with the most substantial decreases in knee and shoulder pain. For instance, knee pain scores decreased from 3.10 ± 2.61 to 1.81 ± 1.69. The overall mean pain rating significantly dropped from 1.72 ± 0.88 pre-intervention to 1.00 ± 0.50 post-intervention, demonstrating the effectiveness of the physiotherapy exercises.

Conclusion: The study confirms the high prevalence of musculoskeletal disorders (MSDs) among handloom weavers and demonstrates the significant impact of physiotherapy interventions in alleviating pain. Implementing regular physiotherapy exercises can substantially improve the well-being and productivity of handloom weavers, ensuring the sustainability of this vital cultural and economic industry. The results advocate for policy changes and increased support for ergonomic and health interventions in the handloom sector.

## Introduction

The handloom weaving industry plays a vital role in developing countries, where traditional weaving techniques remain widely practiced. This industry holds a crucial position in the informal sector, particularly in South Asia, providing livelihoods to millions of people. In India, the handloom sector is deeply rooted in history and tradition, showcasing exceptional craftsmanship that preserves the nation's rich cultural heritage. Indian artisans are globally renowned for their expertise in hand spinning, weaving, and printing, with their products being symbols of elegance and quality [1]. Handloom operations are predominantly household-based, involving collective efforts from various family members. These activities are dispersed throughout numerous towns and villages, facilitating the transmission of skills from one generation to another. The industry sustains more than three million individuals through direct employment and related activities, positioning it as the second leading job provider in rural regions following agriculture [2]. The Handloom Census documents 31.45 lakh households involved in handloom activities across 31 states and union territories. The majority of these weaving households are concentrated in rural regions, with significant numbers in Assam, West Bengal, Manipur, and Tamil Nadu [3].

Despite its cultural and economic significance, the handloom sector faces numerous challenges. Workers frequently endure extended work hours and receive meager pay, lacking job stability or access to social security benefits [4]. The physically demanding nature of the job involves manual sorting of raw materials, carding, spinning, dyeing, and weaving, exposing workers to noise, dust, and repetitive movements that require a high attention to detail. Weaving generally requires repetitive motions of all four limbs, involving the use of pedals and shuttles, resulting in extended durations of static and awkward postures. These working conditions contribute to the high prevalence of musculoskeletal disorders (MSDs) among handloom weavers [5]. Musculoskeletal morbidity is highly prevalent among handloom weavers globally. Various studies report that the prevalence of MSDs among handloom weavers can be as high as 60%-80% in some regions. Frequently cited ailments include discomfort in the lower back, pain in the neck and shoulders, and issues like carpal tunnel syndrome and tendonitis in the hands and wrists. These ailments are primarily caused by poor ergonomic conditions, repetitive motions, prolonged working hours, and inadequate workstation design. The lack of awareness and training on proper ergonomic practices exacerbates the problem [6,7].

Work-related musculoskeletal disorders (WMSDs) rank high among the most financially burdensome occupational afflictions due to their significant impact on both the health and efficiency of workers. The World Health Organization (WHO) estimates that approximately 1.71 billion individuals worldwide suffer from MSDs, making them the primary cause of years lived with disability (YLDs) globally. In India, MSDs represent over 33% of all newly documented work-related illnesses [8]. Addressing the high prevalence of WMSDs among handloom weavers requires a multifaceted approach, including ergonomic improvements, training and education, health support services, and policy advocacy. Implementing effective interventions, such as ergonomic practices and physiotherapy training, can enhance the well-being and productivity of handloom weavers. Promoting a healthier and safer workspace is essential to sustaining this important craft and ensuring its continued contribution to the cultural and economic fabric of developing countries [9].

This study aims to estimate the prevalence of musculoskeletal morbidity and evaluate the impact of remedial exercise training on WMSDs among handloom weavers. By focusing on targeted interventions, this research seeks to address the high burden of WMSDs and improve the health outcomes and productivity of this vital workforce.

## Materials and methods

This study employs a quasi-experimental design, utilizing a pre- and post-test approach, conducted over a period of 12 months from June 2023 to June 2024 in Kanchipuram. The study population includes all handloom weavers in the area, with specific inclusion criteria: weavers aged over 18 years, both male and female, who are full-time workers with at least one year of experience, and who have provided consent for participation in the intervention. Weavers who did not consent, those with permanent physical disabilities, or those who were part-time weavers were excluded.

The study was designed to test the hypothesis that remedial exercise training would have a significant impact on work-related musculoskeletal morbidity among the weavers. The sample size was calculated using a single-group pre- and post-test design, with Cohen’s d set at 1.5 and a power estimation done using G*Power software (Universität Düsseldorf, Germany). The initial sample size was calculated as 110, and to account for potential nonresponse, 10% additional participants were included, bringing the total to 121 handloom weavers. A multistage sampling method was adopted. In the first stage, four major handloom weaver cooperative societies were selected through convenient sampling out of 54 societies. In the second stage, 121 participants were randomly selected from a sampling frame of 1,175 weavers within these societies using simple random sampling.

Ethical approval for the study was obtained from the Institutional Ethics Committee of Saveetha Medical College and Hospital (104/06/2023/IEC/SMCH), with the trial registered under CTRI/2024/02/062757. Data collection was performed using a pretested, semi-structured questionnaire validated by experts from the Departments of Community Medicine and Physiotherapy. The study was conducted in three phases: pre-interventional, interventional, and post-interventional. During the pre-interventional phase, baseline data was collected from the weavers using face-to-face interviews asking for sociodemographic details (Table 6) and standardized tools like the Nordic questionnaire (Figure 2) for MSD prevalence and the numerical pain rating scale (Figure 3) for pain assessment have been attached in the appendices.

In the intervention phase, physiotherapy sessions were provided three days a week for three months, with each session lasting 30 minutes. The intervention was designed and supervised by a registered physiotherapist and monitored by the principal investigator. The exercise protocol was based on the physiotherapy curriculum from Essentials of Orthopedic and Applied Physiotherapy [10]. Physiotherapy exercise module have been attached in the appendices (Table 7). Following the intervention phase, the impact was evaluated using the same pain rating scale to measure changes in pain intensity.

Data entry and analysis were performed using IBM SPSS Statistics for Windows, Version 26 (Released 2019; IBM Corp., Armonk, New York, United States). Descriptive statistics were computed for background variables, while the mean and standard deviation were used for numerical data. Pre- and post-intervention pain scores were compared using paired t-tests, with the chi-square test employed to determine the significance of categorical variables. Odds ratios were calculated to assess the strength of association between study variables and musculoskeletal pain in various body parts. A p-value of less than 0.05 was considered statistically significant, with a 95% confidence interval applied to the results.

## Results

A quasi-experimental study was done among the handloom weavers of Kanchipuram. Among the 121 handloom weavers recruited, an initial assessment was done followed by providing physiotherapy exercises. Baseline data was collected for 121 study participants. With no loss to follow-up, intervention was provided for all 121 handloom weavers. After providing intervention for about three months the parameters were reassessed, and a comparison was made between the pre- and post-intervention.

Table 1 presents the sociodemographic characteristics of the study participants. The mean age of the participants was 54 ± 11 years. The age distribution showed that 60 participants (49.6%) were aged 54 years or younger, while 61 participants (50.4%) were older than 54 years. Regarding gender, the majority of the sample was male, with 85 male participants (70.2%) and 36 female participants (29.8%). Marital status revealed that 119 participants (98.3%) were married, with only two participants (1.7%) reporting being unmarried. The educational status of the study participants, classified according to the Kuppuswamy classification, indicated that 50 participants (41.3%) were illiterate, 59 participants (48.8%) had completed primary education, 10 participants (8.3%) had attained higher secondary education, and one participant each (0.8%) had obtained a diploma or a graduate degree.

**Table 1:** Distribution of sociodemographic characteristics of the study participants

S. no.	Variables		n (%)
1	Age (years)	≤54	60 (49.6)
		>54	61 (50.4)
2	Gender	Male	85 (70.2)
		Female	36 (29.8)
3	Marital status	Married	119 (98.3)
		Unmarried	2 (1.7)
4	Education	Illiterate	50 (41.3)
		Primary	59 (48.8)
		Higher secondary	10 (8.3)
		Diploma	1 (0.8)
		Graduate	1 (0.8)

**Figure 1:**
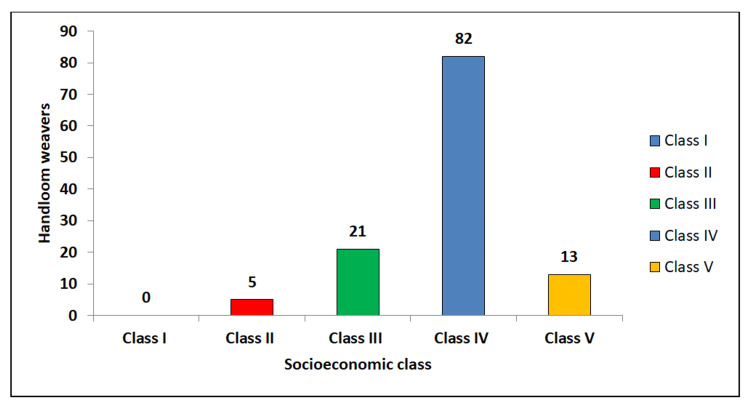
Socioeconomic status among study participants

**Figure 2:**
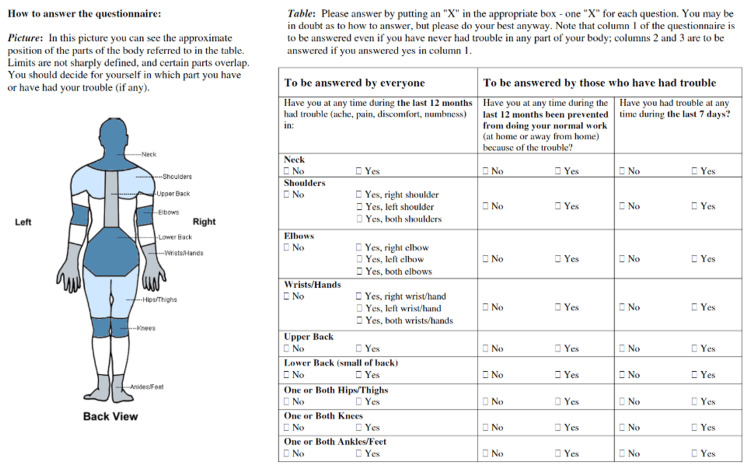
Nordic musculoskeletal questionnaire

**Figure 3:**
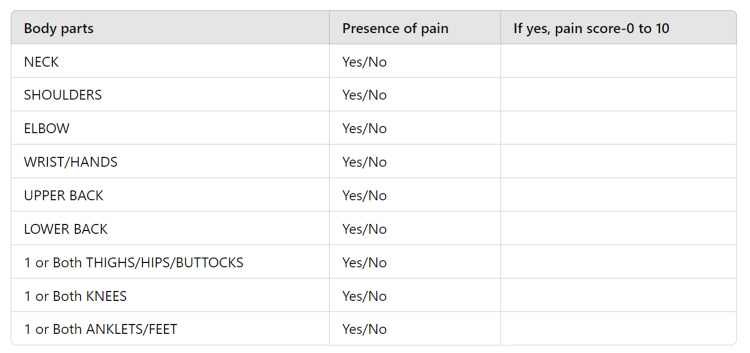
Numerical pain rating scale

The socioeconomic status of the study participants was assessed using the Modified Brahm Govind (BG) Prasad classification (Figure 1). Notably, none of the weavers were categorized under Class I (upper class). In Class II (upper middle class), there were five weavers (4.1%). Class III (middle class) included 21 weavers (17.4%). The majority of the weavers, 82 participants (67.8%), fell into Class IV (lower middle class). Finally, Class V (lower class) included 13 weavers (10.7%).

Table 2 presents the prevalence of musculoskeletal pain reported in various body parts over the past year and in the last seven days. Data were collected using the Standardized Nordic Musculoskeletal Questionnaire. Over the past year, the highest incidences of pain were reported in the knees, with 75 individuals (62%), followed by the ankle/foot region with 66 individuals (54.5%). The lowest prevalence of pain was observed in the elbow, reported by 18 individuals (14.9%), and in the wrists/hands, reported by 21 individuals (17.4%). In the last seven days, knee pain remained prevalent, with 61 individuals (50.4%) reporting discomfort, and ankle/foot pain was reported by 58 individuals (47.9%). Both the elbow and wrists/hands had 18 individuals (14.9%) reporting pain in the last seven days. Conversely, a significant number of participants reported no pain in the elbow (103 individuals, 85.1%) and wrists/hands (100 individuals, 82.6%).

**Table 2:** Prevalence of musculoskeletal pain in each of the body parts using Nordic musculoskeletal questionnaire

History of pain	Body parts								
	Neck n (%)	Shoulder n (%)	Elbow n (%)	Wrists/hands n (%)	Upper back n (%)	Lower back n (%)	Thighs/hips/buttocks n (%)	Knees n (%)	Ankles/feet n (%)
1 year	34 (28.1)	59 (48.8)	18 (14.9)	21 (17.4)	36 (29.8)	47 (38.8)	28 (23.1)	75 (62)	66 (54.5)
7 days	24 (19.8)	51 (42.1)	18 (14.9)	18 (14.9)	28 (23.1)	39 (32.2)	23 (19)	61 (50.4)	58 (47.9)
No pain	87 (71.9)	62 (51.2)	103 (85.1)	100 (82.6)	85 (70.2)	74 (61.2)	93 (76.9)	46 (38)	55 (45.5)

Table 3 presents the quantification of pain using the numerical pain rating scale, comparing mean pain scores and their standard deviations (SD) across various body parts before and after the intervention. Pre-intervention, the highest pain scores were reported in the knees (3.10 ± 2.61) and ankles/feet (2.91 ± 2.80), while the lowest scores were observed in the elbow (0.76 ± 1.98) and wrists/hands (0.90 ± 2.05). Post-intervention, all body parts showed a reduction in pain scores. Notably, the knees (1.81 ± 1.69) and shoulders (1.19 ± 1.60) exhibited substantial reductions in pain. The least change was noted in the elbow (0.60 ± 1.58). These findings suggest a significant overall reduction in pain across all body parts following the intervention, indicating its effectiveness.

**Table 3:** Quantification of pain in each of the body parts using the numerical pain rating scale SD: Standard deviation

	Neck	Shoulder	Elbow	Wrists/hands	Upper back	Lower back	Thighs/hips/buttocks	Knees	Ankles/feet
Pre-intervention pain score (mean + SD)	1.21 ± 1.95	2.33 ± 2.55	0.76 ± 1.98	0.90 ± 2.05	1.28 ± 2.04	1.90 ± 2.49	1.08 ± 2.01	3.10 ± 2.61	2.91 ± 2.80
Post-intervention pain score (mean + SD)	0.86 ± 1.47	1.19 ± 1.60	0.60 ± 1.58	0.49 ± 1.20	0.45 ± 0.86	1.05 ± 1.54	0.78 ± 1.46	1.81 ± 1.69	1.75 ± 1.80

Table 4 presents the paired t-test results for the numerical pain rating scale, demonstrating significant reductions in pain scores post-intervention across various body parts. The mean pain score for the neck decreased from 1.21 to 0.86, with a mean difference of 0.34. Shoulder pain reduced from 2.33 to 1.19, yielding a mean difference of 1.14. Elbow pain scores dropped from 0.76 to 0.60, with a mean difference of 0.16. Pain in the wrist/hand decreased from 0.90 to 0.49, with a mean difference of 0.40. Upper back pain reduced from 1.28 to 0.45, with a mean difference of 0.82, while lower back pain decreased from 1.90 to 1.05, with a mean difference of 0.84. Thigh/hip/buttock pain reduced from 1.08 to 0.78, yielding a mean difference of 0.29. Knee pain scores dropped significantly from 3.10 to 1.81, with a mean difference of 1.28, and ankle/feet pain decreased from 2.91 to 1.75, with a mean difference of 1.16. These findings indicate that the intervention significantly reduced pain across all examined body parts, with a p-value of <0.01 for all comparisons.

**Table 4:** Impact of remedial exercise training on each of the body parts using numerical pain rating scale *p-value < 0.05: significant (paired t-test)

Outcome measure	Body parts	Intervention phase	Mean	Standard deviation	Mean difference	t-value	p-value
Numerical pain rating scale	Neck	Pre	1.21	1.95	0.34	6.66	<0.01*
		Post	0.86	1.47			
	Shoulder	Pre	2.33	2.55	1.14	10.28	<0.01*
		Post	1.19	1.60			
	Elbow	Pre	0.76	1.98	0.16	4.18	<0.01*
		Post	0.60	1.58			
	Wrists/hands	Pre	0.90	2.05	0.40	4.90	<0.01*
		Post	0.49	1.20			
	Upper back	Pre	1.28	2.04	0.82	7.02	<0.01*
		Post	0.45	0.86			
	Lower back	Pre	1.90	2.49	0.84	8.52	<0.01*
		Post	1.05	1.54			
	Thighs/hips/buttocks	Pre	1.08	2.01	0.29	5.58	<0.01*
		Post	0.78	1.46			
	Knees	Pre	3.10	2.61	1.28	13.68	<0.01*
		Post	1.81	1.69			
	Ankle/feet	Pre	2.91	2.80	1.16	11.67	<0.01*
		Post	1.75	1.80			

Table 5 evaluates the impact of remedial exercise training using a paired t-test. The analysis showed that the mean pain rating before the intervention was 1.72 (SD = 0.88). Post-intervention, the mean pain rating significantly decreased to 1.00 (SD = 0.50). The mean difference between pre- and post-intervention pain ratings was 0.72. This difference was statistically significant, with a t-value of 5.08 and a p-value of <0.001. These findings suggest that the intervention was highly effective in reducing pain levels, with a very low probability that the observed change occurred by random chance.

**Table 5:** Overall impact of remedial exercise training using numerical pain rating scale *p-value < 0.05: significant (paired t-test)

Outcome measure	Phase	Mean	Standard deviation	Mean difference	t-value	p-value
Numerical pain rating scale	Pre-intervention	1.72	0.88	0.72	5.08	<0.001*
	Post-intervention	1.00	0.50			

## Discussion

A study investigating the prevalence of MSDs among handloom weavers in Kanchipuram found notable rates of pain across various body regions. Neck pain was prevalent in 38.8% of weavers, shoulder pain in 48.8%, and elbow pain in 14.9%. These figures are contrasted with findings from a study by Satheeshkumar and Krishnakumar in Kerala, which reported higher neck pain at 44.32%, but lower shoulder and elbow pain at 35.46% and 51.52%, respectively. Differences in ergonomic practices, equipment design, and workplace layout between regions may contribute to these variations in MSD prevalence [11]. The study also highlighted the prevalence of pain in other body parts among Kanchipuram weavers, including 17.4% in the wrist/hand, 29.8% in the upper back, and 38.8% in the lower back. In comparison, Satheeshkumar and Krishnakumar found slightly lower wrist/hand pain at 14.96% but higher rates of upper back and lower back pain at 38.78% and 61.77%, respectively. These differences suggest that regional factors, such as access to healthcare, awareness of MSDs, and early intervention, can significantly influence reported pain prevalence among handloom weavers [11].

Additionally, the study in Kanchipuram identified high rates of pain in the thighs/hips/buttocks (23.1%), knees (62%), and ankles/feet (54.5%). Contrastingly, the Kerala study reported higher thighs/hips/buttocks pain at 53.74% but lower knee and ankles/feet pain at 34.90% and 22.16%, respectively. The repetitive and physically demanding nature of handloom weaving, including prolonged standing and awkward postures, likely contributes to these high pain rates. Variations in loom types, weaving techniques, and the intensity of work across regions also play a crucial role in the differing MSD prevalence rates observed between the two studies [11].

The physiotherapy intervention resulted in a significant reduction in pain scores across all body parts, with the most substantial improvements seen in the knees and shoulders. Before the intervention, the average pain rating was 1.72 (SD = 0.88), which decreased to 1.00 (SD = 0.50) post-intervention. This change, with a mean difference of 0.72 (p < 0.001), highlights the effectiveness of physiotherapy exercises in alleviating musculoskeletal pain among handloom weavers. Similarly, a study done by Yogeshwaran implies that both the control group and the experimental group have significant improvements in back pain and reduced disability induced by back pain followed by physiotherapy intervention among handloom weavers [12]. Similarly, a study by Varghese in India utilized the numeric pain rating scale to compare pre-treatment and post-treatment pain scores between control and experimental groups. The pre-treatment mean pain scores were 6.33 for the control group and 5.93 for the experimental group. Post-treatment, the mean pain scores were 4.27 for the control group and 6.33 for the experimental group. This indicates an increase of 0.4 units in pain for the control group and a decrease of 2.06 units in pain for the experimental group, demonstrating a significant reduction in pain for those in the experimental group after physiotherapy intervention among handloom weavers [13].

These findings underscore the effectiveness of physiotherapy exercises in managing musculoskeletal pain among handloom weavers. The targeted exercises likely improved muscle strength, flexibility, and posture, essential for reducing the risk of MSDs and improving overall musculoskeletal health. This study, along with supporting literature, highlights the potential benefits of implementing regular physiotherapy programs in handloom settings to enhance weaver’s health and productivity. A limitation of the study was the lack of long-term follow-up, which would have been necessary to assess the sustainability of the intervention's effects on musculoskeletal pain. Future studies with extended follow-up are needed to evaluate the long-term impact of the intervention more comprehensively.

## Conclusions

This quasi-experimental study sheds light on the prevalence of musculoskeletal pain among handloom weavers in Kanchipuram, Tamil Nadu, and evaluates the effectiveness of physiotherapy interventions in reducing such pain. This study underscores the necessity of incorporating regular physiotherapy to improve the well-being and productivity of handloom weavers. The significant pain reductions observed across all body parts in this study highlight the importance of physiotherapy interventions in managing musculoskeletal pain and improving the quality of life for handloom weavers.

## Appendices

**Table 6:** Sociodemographic details

S. no.	Variables	Response
1	Name	
2	Age	
3	Gender	
4	Marital status	Married/unmarried
5	Educational status	Illiterate/primary/higher secondary/diploma/graduate
6	Socioeconomic status	Class I/II/III/IV/V

**Table 7:** Physiotherapy exercise module

Area	Exercise	Instructions
Neck	Neck flexion	Sit or stand with your back straight and shoulders relaxed. Slowly lower your chin toward your chest, feeling a gentle stretch in the back of your neck. Hold the stretch for 15-30 seconds, while breathing deeply and evenly. Return to the starting position slowly. Repeat the movement 3-5 times
	Neck extension	Sit or stand with your back straight and shoulders relaxed. Gently tilt your head backward, looking up toward the ceiling. Hold the stretch for 15-30 seconds, feeling a gentle stretch in the front of your neck. Return to the starting position slowly. Repeat the movement 3-5 times
	Isometric side flexion	Sit or stand tall, shoulders relaxed. Keep head neutral, facing forward. Place hand near ear on one side. Push head gently toward shoulder. Resist with neck muscles, hold for 5-10 seconds. Relax and return to start. Repeat on opposite side. Perform 3-5 reps on each side
Shoulder	Overhead triceps stretch	Stand tall or sit. Lift one arm overhead, bending it at the elbow. Reach your hand down your back, toward the middle of your shoulder blades. With your other hand, gently grasp the elbow of your bent arm. Gently pull your elbow toward the midline of your body, feeling a stretch along the back of your upper arm (triceps). Hold the stretch for 15-30 seconds, while breathing deeply and evenly. Switch arms and repeat the stretch on the other side. Perform 2-3 repetitions on each side
	Cross-body shoulder stretch	Stand tall or sit upright in a chair. Extend one arm across your body at shoulder height. Use your other hand to gently press the extended arm toward your chest until you feel a stretch in the back of your shoulder. Hold the stretch for 15-30 seconds, while breathing deeply and evenly. Switch arms and repeat the stretch on the other side. Perform 2-3 repetitions on each side
Elbow	Forearm pronation and supination	Sit with your elbow bent at 90 degrees and your forearm resting on a table or your thigh. Rotate your forearm so your palm faces up (supination), then rotate it so your palm faces down (pronation). Repeat 10-15 times
Wrists/hands	Wrist flexor stretch	Extend one arm in front of you with your palm facing down. Use your other hand to gently pull your fingers back toward your body, feeling the stretch along the underside of your forearm. Hold for 15-30 seconds. Switch arms and repeat
	Wrist extensor stretch	Extend one arm in front of you with your palm facing down. Use your other hand to gently press your fingers down toward the ground, feeling the stretch along the top of your forearm. Hold for 15-30 seconds. Switch arms and repeat
Upper back	Pectoralis stretch	Stand in a doorway or corner of a room. Place one hand on the doorframe or wall at shoulder level. Position your elbow at a 90-degree angle. Slowly lean forward, keeping your spine straight, until you feel a stretch in the front of your chest and shoulder. Hold this position for 15-30 seconds while breathing deeply. Switch sides and repeat
Lower back	Pelvic bridge	Lie on your back with knees bent and feet flat. Engage core and lift hips toward the ceiling. Squeeze glutes at the top. Hold briefly, avoiding arching the lower back. Lower hips down slowly while keeping core engaged. Aim for 10-15 controlled repetitions, focusing on proper form
	Prone press-up	Lie on your stomach with your hands placed flat on the ground near your shoulders. Push through your hands to lift your upper body off the ground, arching your back gently. Keep your hips and pelvis in contact with the ground. Hold the extended position for a few seconds, then lower back down. Repeat for several repetitions, gradually increasing the range of motion as tolerated
	Cat and camel stretch	Begin on your hands and knees, with your wrists aligned under your shoulders and your knees under your hips. Inhale as you arch your back upward like a cat, tucking your chin toward your chest and rounding your spine. Exhale as you move into the camel position, lowering your belly toward the floor, lifting your head and tailbone, and arching your back downward. Flow smoothly between the cat and camel positions, moving with your breath. Repeat for several repetitions, focusing on the fluidity of movement and the stretch in your spine
Thighs/hips/buttocks	Straight leg raise	Lie Flat On your back, legs straight, arms by your sides. Tighten your abdominal muscles. Lift one leg straight up to a 45-degree angle, keeping the other leg on the ground. Pause for a few seconds. Slowly lower the leg back down. Do 10-15 repetitions on each leg
Knee	Isometric knee exercise	Lie down on your back, place a foam roller or rolled-up towel under one knee. The other leg can be straight or bent. Press the back of your knee into the roller to engage your quadriceps. Maintain the pressure for 5-10 seconds. Release and rest for a few seconds. Perform 10-15 repetitions on each leg
Ankles/feet	Toe raise	Stand tall with your feet hip-width apart. Lift your toes off the ground while keeping your heels planted. Hold for a moment, then slowly lower your toes back to the ground. Repeat for the desired number of repetitions
	Heel raise (calf raise)	Stand tall with your feet hip-width apart. Lift your heels off the ground, rising up onto your toes. Hold for a moment at the top, then slowly lower your heels back to the ground. Repeat for the desired number of repetitions
